# Maternal Factors, Breast Anatomy, and Milk Production During Established Lactation—An Ultrasound Investigation

**DOI:** 10.3390/jimaging11090313

**Published:** 2025-09-12

**Authors:** Zoya Gridneva, Alethea Rea, David Weight, Jacki L. McEachran, Ching Tat Lai, Sharon L. Perrella, Donna T. Geddes

**Affiliations:** 1 School of Molecular Sciences, The University of Western Australia, Crawley, WA 6009, Australia; jacki.mceachran@uwa.edu.au (J.L.M.); ching-tat.lai@uwa.edu.au (C.T.L.); sharon.perrella@uwa.edu.au (S.L.P.); donna.geddes@uwa.edu.au (D.T.G.); 2 ABREAST Network, Perth, WA 6000, Australia; 3 UWA Centre for Human Lactation Research and Translation, Crawley, WA 6009, Australia; 4 Mathematics and Statistics, Murdoch University, Murdoch, WA 6150, Australia; alethea.rea@murdoch.edu.au (A.R.); david.weight@murdoch.edu.au (D.W.)

**Keywords:** ultrasound, glandular tissue, milk ducts, breast anatomy, breastfeeding, lactation, milk production, body composition

## Abstract

Obesity is linked to suboptimal breastfeeding outcomes, yet the relationships between maternal adiposity, breast anatomy, and milk production (MP) have not been investigated. We conducted ultrasound imaging to assess the breast anatomy of 34 lactating women. The amount of glandular tissue (glandular tissue representation (GTR)) was classified as low, moderate, or high. Number and diameters of main milk ducts and mammary blood flow (resistive index) were measured. Women completed a 24 h MP measurement and an obstetric/lactation history questionnaire. Body composition was measured with bioimpedance spectroscopy. Statistical analysis employed correlation networks. Multiple relationships were revealed, with later menarche correlating with minimal pubertal and pregnancy breast growth. A minimal breast growth was further correlated with lower mammary blood flow during lactation and lower numbers and smaller diameters of main milk ducts, which in turn correlated with a lower MP. Importantly, higher adiposity also correlated with minimal breast growth during pregnancy and low GTR and MP. Several modifiable and non-modifiable maternal factors may be associated with breast development and MP. Antenatal lactation assessment and intervention in high-risk women may ensure they reach their full lactation potential and inform future interventions, such as maintaining healthy adiposity.

## 1. Introduction

Breastfeeding is a major public health and nutrition focus. Breastfeeding is linked to multiple dose-related health benefits for breastfeeding dyads [1,2], including a reduction in obesity risk [3,4,5]. One of the main reasons for the early cessation of breastfeeding is low milk supply (LMS), either perceived or true [6,7]. It is important to understand modifiable and non-modifiable factors that place mothers at risk of LMS [8,9] in order to inform the development of interventions aimed at increasing milk production (MP).

Animal models show a more rapid mammary gland response and more milk during secretory activation with subsequent lactations [10,11], as well as the impairment of lactation performance by obesity via inducing prolactin resistance [12]. In humans, no effect of consecutive lactation on MP was confirmed in fully breastfeeding women [13]. However, maternal obesity has been linked to later secretory activation [14,15,16,17,18], reduced breastfeeding initiation [17,18,19,20], diminished breastfeeding confidence [21], and increased difficulties [22] commensurate with decreased duration of exclusive or any breastfeeding [14,15,17,18,19,20,23]. Further, a recent study reported that increased maternal body mass index (BMI) was associated with low MP (<600 g/24 h [24,25]), whilst larger pre-pregnancy breast volume and breast growth during pregnancy were associated with both a higher BMI and MP [26]. Despite this, the mechanisms and pathways implicated in obesity-related LMS are not well established, and relationships between maternal adiposity, breast anatomy, and MP have not been assessed.

Diagnostic ultrasound imaging of the breast has largely been limited to the non-lactating breast for the detection of pathologies. Studies of the lactating breast including ultrasound investigations are rare, but they help to understand the macro- and micro-anatomy pertaining to MP and removal [1,27]. One of the requirements for successful lactation is sufficient glandular tissue [27]. The conventional understanding of lactational breast anatomy states that glandular tissue is the predominant tissue type, yet it has been shown that glandular tissue may comprise 45–83% of the breast, with wide variability between women [28]. To date, no relationships between MP and the amount of glandular tissue, the number of the milk ducts, or duct diameters have been established [28].

While mammary blood flow doubles during pregnancy [29], blood flow characteristics are rarely measured in the human lactating breast with increased metabolic activity. Only one published study in this area was unable to demonstrate a relationship between mammary blood flow and 24 h MP [30]. The study included participants with normal MP, and in the three participants with significantly reduced mammary blood flow, negligible amounts of milk were synthesized, suggesting there may be a threshold below which MP may be impacted [30]. The average 24 h mammary blood flow to MP ratio in women was approximately 400:1 (L), with high variability between women. Unfortunately, no information on the degree of fullness of the breast or the storage capacity of the breast was available, which could be helpful to determine if the amount of milk in the breast and/or the glandular tissue volume are related to the blood flow. Thus, breast anatomy and mammary blood flow in lactating women should be further investigated to understand relationships with LMS and MP in general.

This pilot study employed ultrasound to elucidate the relationships between (a) the breast anatomy of lactating women, (b) adiposity, (c) medical, obstetric and lactation history, and (d) 24 h MP and milk removal efficacy. A preliminary summary of this study’s results has been presented at the ABREAST Conference 2023 [31].

## 2. Materials and Methods

### 2.1. Participants and Study Design

This is a cross-sectional study of lactating women 1–6 months postpartum who lived in the Perth metropolitan area, Western Australia, and attended a study visit at The University of Western Australia between 2018 and 2020. During the study visit, breast anatomy was assessed with ultrasound imaging. Maternal body composition was measured with bioimpedance spectroscopy. Women completed questionnaires regarding demographic and obstetric details, including age, the onset of menarche, breast growth during puberty and pregnancy, parity, previous lactation duration, birth mode, birth gestation, use of hormonal contraception, and postpartum return of menstruation. Prior to the study visit, women used electronic scales in their home to conduct 24 h MP measurements to enable calculation of total volume, average, and maximum feed volumes and breast storage capacity (24 h MP parameters).

Selection criteria included breastfeeding mothers of singleton term infants between 1 and 6 months postpartum. This study was conducted in accordance with the Declaration of Helsinki and was approved by The University of Western Australia Human Research Ethics Committee (RA/4/1/4492 and RA/4/1/8532). Informed written consent was obtained from all participants.

### 2.2. Ultrasound Examination of the Breast

We conducted ultrasound examination to assess breast anatomy using a Xario 200 ultrasound machine (Canon Medical Systems ANZ, Castle Hill, NSW, Australia), a high-resolution transducer with frequency range of 7.0–12.0 MHz (PLU-1005BT) and Parker ultrasonic gel (Fairfield, NJ, USA). For all measurements, a single breast was randomly selected and scanned prior to expressing milk. All measurements were made at the same venue by the same experienced sonographer with demonstrated good intra- and interrater reliability [30].

**Figure 1:**
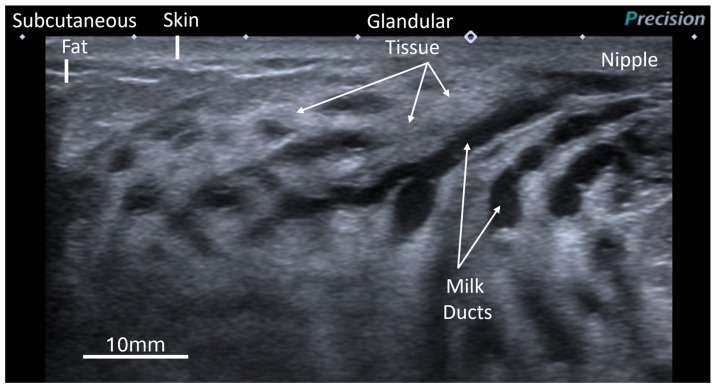
Ultrasound image of the lactating breast showing main milk ducts (dark, hypoechoic) within the breast, under the areola. Glandular tissue is hyperechoic and the fat more hypoechoic compared to the glandular tissue.

The number of main milk ducts and duct diameters beneath the areola were measured as described using the radial clock face method [28]. The milk duct is visualized as a hypoechoic (low echo) tubular structure (Figure 1). As increased milk duct diameters during pumping sessions have previously been linked to increased milk flow rates [32], we calculated the means and sums of duct diameters for the entire breast and also categorized them into the superior and inferior halves of the breast.

Due to the difficult and time-consuming nature of methods used to quantify mammary glandular and adipose tissue [28], a proxy measure of the amount of glandular tissue, glandular tissue representation (GTR), was used in this study. The GTR was classified as low (approximately < 30%), moderate (approximately 30–70%), or high (approximately > 70%). On ultrasound, the skin appears as an echogenic line at the top of the image, whilst the glandular tissue appears as echogenic (hyperechoic) tissue, and the adipose tissue is hypoechoic tissue within the breast (Figure 1) [28].

**Figure 2:**
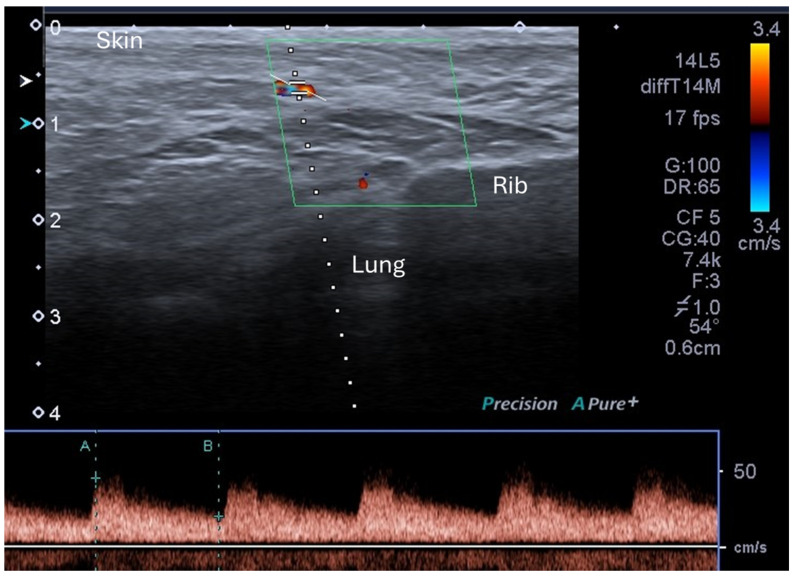
Colour flow Doppler imaging of the internal mammary artery visualised as the coloured vessel within the green box. The colours represent velocity of the blood flow. A, peak systolic velocity; B, end diastolic velocity as measured by pulsed wave doppler sampled within the internal mammary artery.

Blood flow in the dominant branch of the internal mammary artery, which contributes the greater proportion of blood flow to the lactating breast (60–70%) [30,33], was measured with colour Doppler ultrasound (Figure 2), as previously described [30]. Settings were adjusted to optimize the image and Doppler spectrum accordingly for each participant. Measurements made from the Doppler waveforms included peak systolic and peak end diastolic velocities.

The internal mammary arterial resistive index, derived from the maximum and minimum velocities, was calculated using the formula below (Equation (1)), where *RI* is resistive index, *PSV* is the peak systolic velocity, and *EDV* is the end diastolic velocity [34,35]:

(1) RI=(PSV−EDV)/PSV

### 2.3. Anthropometric and Body Composition Measurements

Participants’ body weights were measured using an electronic scale (accuracy ± 0.1 kg; Seca, Chino, CA, USA). Height was self-reported by participants or measured against a calibrated marked wall (accuracy ± 0.1 cm). BMI was calculated as kg/m^2^. The breast volume (cm^3^) was calculated based on both the current bra cup size and band size reported by participants, with reference to an online chart [36].

Body composition was measured with bioelectrical impedance spectroscopy using an Impedimed SFB7 bioelectrical impedance analyser (ImpediMed, Brisbane, QLD, Australia), according to protocols described previously [37], and applying resistivity coefficients for the healthy females [38]. In addition to standard body composition measurements (fat-free mass (FFM), fat mass (FM), and %FM), the height-normalized body composition indices (FFMI, FMI) [39] and FM to FFM ratio (FM/FFM) were calculated.

### 2.4. The 24 h Milk Profile Data Collection

The 24 h MP measurements were performed on average 2.6 ± 4.8 (mean ± SD) weeks prior to the study visit using the test-weighing method [25,40]. Briefly, pre- and post-feed weights of infants as well as weights of milk collection bottles before and after any breast expressions were recorded using electronic scales (±2.0 g; Electronic Baby Weigh Scale, Medela Inc., McHenry, IL, USA) for all feeds/expressions in one 24 h period plus one feed/expression. All expressed milk or feed weights were recorded and reported in grams [41]. The 24 h MP was calculated as the total volume of all breastfeeds and breast expressions during 24 h.

During the 24 h MP measurement, participants collected small (<2.0 mL) milk samples pre- and post-expressions and/or breastfeeds by manual expression into 5 mL polypropylene tubes (P5016SL, Techno Plas Pty Ltd., St Marys, SA, Australia). Samples were stored in the participant’s home freezer until transported to the laboratory, where fat concentration was analyzed using the creamatocrit method [42]. Using the pre- and post-expression fat concentrations and participants’ 24 h MP data, the degree of fullness of the breast (DOF) and percentage of available milk removed (PAMR) at each milk removal session, as well as the breast storage capacity, were calculated for each breast based on the method described previously [43,44]. The 24 h average feed volume, maximum feed volume, breast storage capacity, and average PAMR are reported for the test breast only.

### 2.5. Statistical Analyses

Descriptive statistics are presented as mean ± standard deviation (SD) or median and interquartile range for continuous variables and frequencies/counts and percentages for categorical variables.

Statistical analysis used the correlation networks method [45], which have each variable as a node, and the edges represent the relationships between the variables with colour representing the type of association (positive or negative) and thickness and saturation representing the strength of the association. The 36 variables included some numeric measures (time postpartum, total 24 h MP, test breast 24 h MP, test breast average feed, test breast maximum feed, test breast storage capacity, test breast average PAMR, mammary artery resistive index, test breast total ducts number, ducts number in inferior half of the test breast, ducts number in superior half of the test breast, mean duct diameter in the test breast, mean duct diameter in inferior half of the test breast, mean milk duct diameter in superior half of the test breast, total sum of duct diameters, sum of duct diameters in inferior half of the test breast, sum of duct diameters in superior half of the test breast, maternal age at menarche, maternal age at the study visit, breast cup volume, body weight, BMI, FFM, FFMI, FM, %FM, FMI, FM/FFM, and duration of previous lactations) and some categorical measures (GTR, parity, hormonal contraception use, menstrual cycle commenced (yes), puberty breast growth, pregnancy breast growth, and birth mode), and, as such, a mix of Polychoric, polyserial, and Pearson correlations were used as implemented in cor_auto from the qgraph package in R (version 4.4.0) [46]. The results are reported as *r* (correlation coefficient or correlation strength) and *p*-value in the tables, and data visualization was achieved using heat maps. Due to the large number of edges in the network, only statistically significant edges were visualized (significance level 0.05, *p*-values were not adjusted). Missing data were addressed using available case analysis (pairwise deletion) to preserve the sample sizes and to have a higher efficiency whilst avoiding bias and loss of precision [47].

Additionally, the correlations were visualized with heat maps completed in Excel using the correlation coefficients and displaying the correlations between multiple variables as a two-colour gradient. The colour of each cell corresponds to the magnitude of the cell amount with the darker colours representing stronger correlations and colours themselves representing the direction of correlation using the diverging colour palette and enabling clustering [48].

## 3. Results

### 3.1. Participants’ Demographics and Obstetric History

Thirty-four lactating women participated in this study (Table 1). Most participants (90%) identified as Australian. Body composition was measured at 4.0 ± 1.2 months postpartum and on average 2.5 ± 1.2 weeks after the 24 h MP measurement. More than half of the participants (60.6%) had an overweight or obese BMI: normal (BMI 18.5–24.9, *n* = 13, and 39.4%; %FM 21–32.9%, *n* = 3, and 9.1%), overweight (BMI 25–29.9, *n* = 13, and 39.4%; %FM 33–38.9%, *n* = 18, and 54.5%), and with obesity (BMI > 30, *n* = 7, and 21.2%; %FM > 39%, *n* = 12, and 36.4%) [49].

Participants’ reproductive and obstetric history are presented in Table 2. More than half of the participants were primiparous and had a vaginal birth.

### 3.2. Breast Anatomy Parameters

The breast ultrasound examination showed that most of the milk ducts (70%) were positioned in the inferior half of the breast (Table 3). The majority of participants (56%) had a high GTR, with 29% having moderate and 15% low GTR.

### 3.3. The 24 h Milk Production Parameters

The 24 h MP was measured at 3.5 ± 1.2 months postpartum and was 809 ± 235 g (min–max: 321–1274 g; Table 4); eight participants (24%) had a LMS defined as <600 g/24 h [24,25].

### 3.4. Correlation Matrix Results

**Figure 3:**
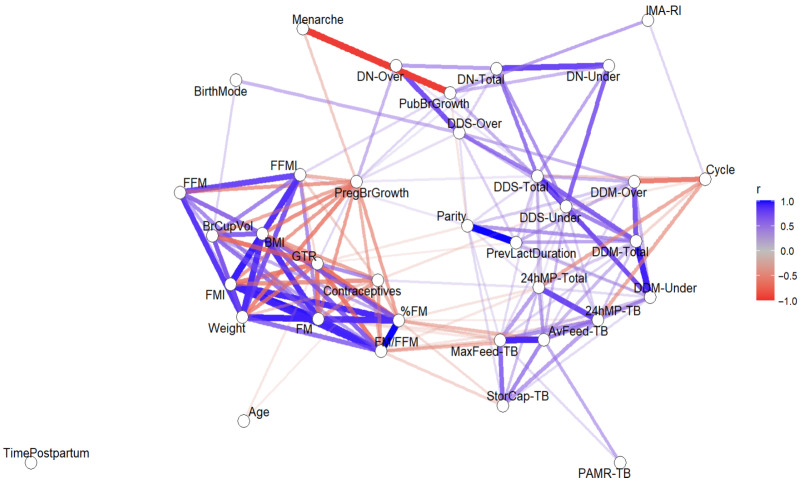
The correlation matrix results (*n* = 34) for illustrative purposes. The proximity and thickness of the lines represent the strength of correlations, and colours represent both the significance and direction of correlations: dark blue—strong positive, dark red—strong negative. 24hMP-TB, test breast 24 h milk production (g); 24hMP-Total, total 24 h milk production (g); Age, maternal age (years); AvFeed-TB, test breast average feed (g); BMI, body mass index (kg/m^2^); BirthMode, birth mode (vaginal); BrCupVol, breast cup volume (cm^3^); Contraceptives, hormonal contraception use (yes); Cycle, cycle commenced (yes); DDM-Over, mean milk duct diameter in superior half of the test breast (mm); DDM-Total, mean duct diameter in the test breast (mm); DDM-Under, mean duct diameter in inferior half of the test breast (mm); DDS-Over, sum of duct diameters in superior half of the test breast (mm); DDS-Total, total sum of duct diameters (mm); DDS-Under, sum of duct diameters in inferior half of the test breast (mm); DN-Over, ducts number in superior half of the test breast; DN-Total, total ducts number; DN-Under, ducts number in inferior half of the test breast; FFM, maternal fat-free mass (kg); FFMI, fat-free mass index (kg/m^2^); FM, fat mass (kg); %FM, percentage fat mass (%); FMI, fat mass index (kg/m^2^); FM/FFM, fat mass to fat-free mass ratio; GTR, glandular tissue representation (low (<30%), moderate (30–70%), or high (>70%)); IMA-RI, internal mammary artery resistive index; MaxFeed-TB, test breast maximum feed (g); Menarche, maternal age at menarche (years); PAMR-TB, test breast average percentage of available milk removed (%); Parity, parity; PregBrGrowth, pregnancy breast growth (yes); PrevLactDuration, duration of previous lactations (months); PubBrGrowth, puberty breast growth (yes); StorCap-TB, test breast storage capacity (g); TimePostpartum, time postpartum (months); Weight, maternal body weight (kg).

**Figure 4:**
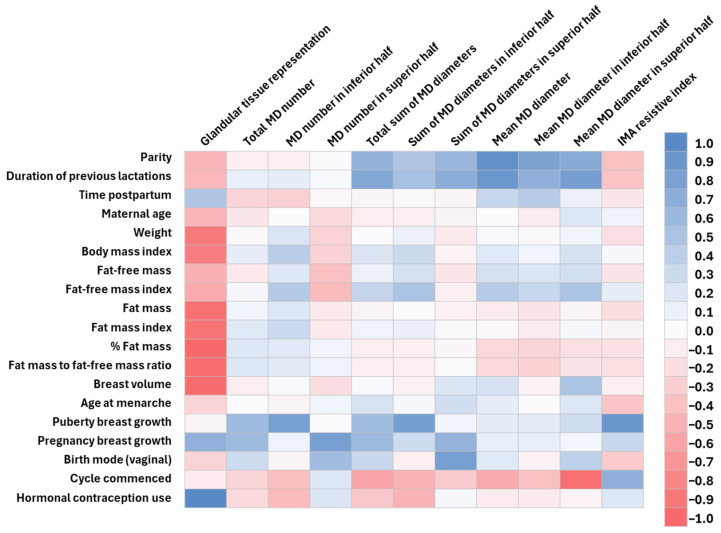
The correlation coefficients between breast anatomy parameters and maternal characteristics including adiposity. IMA, intra mammary artery; MD, milk ducts. Dark blue represents strong positive and dark pink strong negative correlations.

**Figure 5:**
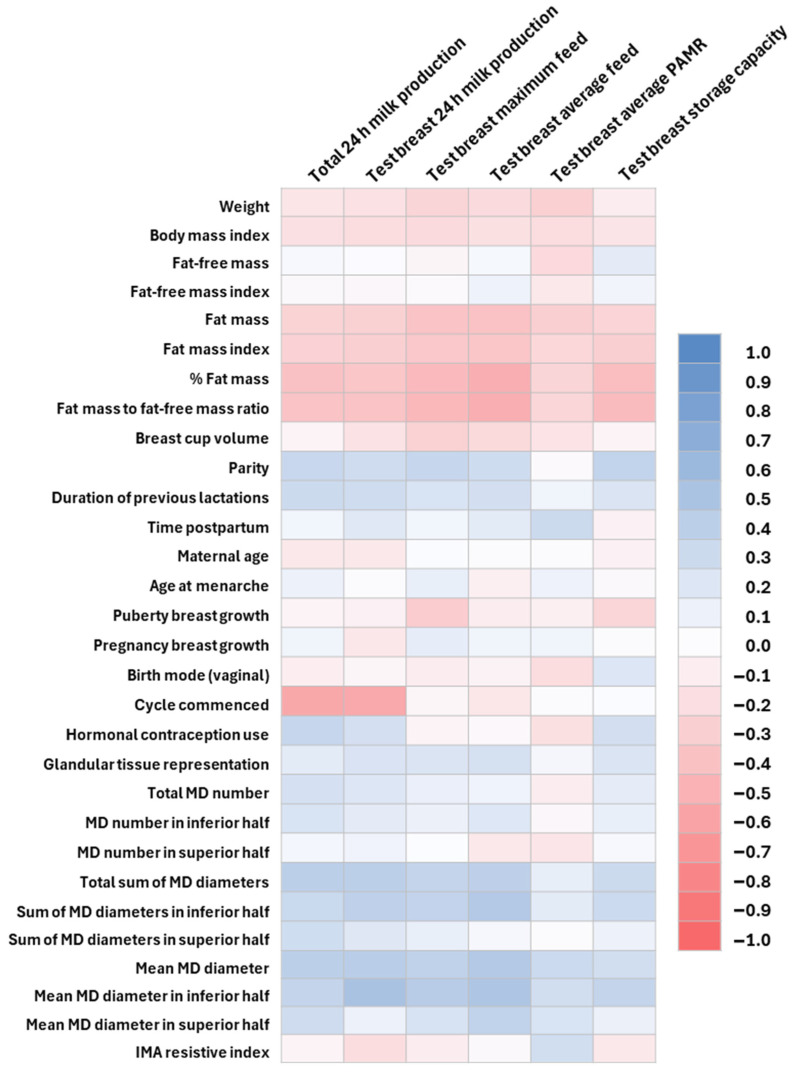
The correlation coefficients between 24 h milk production parameters and maternal characteristics, maternal adiposity, and breast anatomy parameters. IMA, intra mammary artery; MD, milk ducts; and PAMR, percentage of available milk removed. Dark blue represents strong positive and dark pink strong negative correlations.

**Figure 6:**
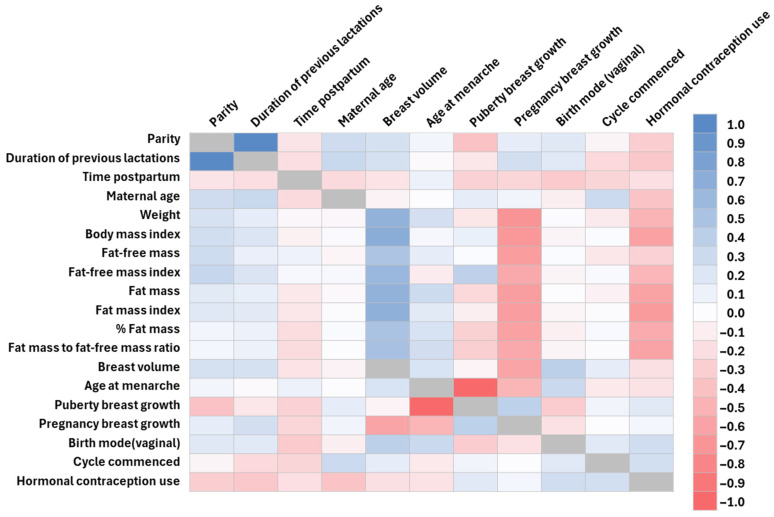
The correlation coefficients between maternal characteristics and maternal body composition parameters. Dark blue represents strong positive and dark pink strong negative correlations. Grey colour—not applicable.

The correlation matrix results (Figure 3) are presented in more detail in Figure 3, Figure 4, Figure 5 and Figure 6 and Table A1, Table A2, Table A3, Table A4 and Table A5. There were no significant correlations with the time postpartum.

#### 3.4.1. Breast Development and Anatomy

There were multiple strong positive correlations between the number of main milk ducts and the sum of diameters measured in both halves of the breast combined and in the superior and inferior halves; however, the mean of the duct diameters did not correlate with the numbers of ducts (Table A6).

The number of main milk ducts was positively correlated with breast growth both during puberty and in pregnancy (Figure 4, Table A1 and Table A2). The mean duct diameters were negatively correlated with the postpartum return of the menstrual cycle. The sums of the duct diameters were positively correlated with the duration of previous lactations and parity, breast growth during puberty and pregnancy, and vaginal birth and negatively with the return of the menstrual cycle and hormonal contraception use.

GTR was positively correlated with breast growth during pregnancy and the use of hormonal contraception and negatively with maternal age, parity, and duration of previous lactations (Figure 4, Table A1 and Table A2). The internal mammary artery resistive index was positively correlated with breast growth during puberty and the return of the menstrual cycle. Milk duct measurements and the resistive index were not correlated with any body composition parameters; however, the GTR was negatively correlated with weight, BMI, FFM, FFMI, FM, FMI, %FM, FM/FFM, and breast volume and positively with breast growth during pregnancy and hormonal contraception use.

#### 3.4.2. The 24 h Milk Production

There were multiple strong positive correlations between the 24 h MP parameters, including the total 24 h MP as well as the test breast’s 24 h MP, maximum feed volume, average feed volume, PAMR, and breast storage capacity (Table A7).

The relationships of 24 h MP with maternal factors, body composition, and breast anatomy are presented in Figure 5 and Table A3. The total 24 h MP was positively correlated with parity and hormonal contraception use and negatively with the return of the menstrual cycle. The test breast’s 24 h MP was negatively correlated with the return of the menstrual cycle. The test breast maximum feed and breast storage capacity were positively correlated with parity.

The total 24 h MP and test breast’s 24 h MP, maximum feed volume, and average feed volume were positively correlated with the means and sums of the duct diameters and negatively with maternal %FM and FM/FFM (Figure 5, Table A3).

The breast storage capacity was positively correlated with mean duct diameters in the inferior half of the breast and negatively with maternal %FM and FM/FFM. The PAMR was not significantly correlated with any maternal characteristics, body composition, or breast anatomy parameters. Breast volume, GTR, and the number of main milk ducts had no relationships with 24 h MP parameters (Figure 5, Table A3).

#### 3.4.3. Maternal Characteristics and Body Composition

There were multiple strong positive correlations between maternal body composition parameters (Table A8).

All maternal body composition parameters were positively correlated with breast volume and negatively correlated with breast growth during pregnancy. All maternal body composition parameters were negatively correlated with hormonal contraception use apart from FFM. The FFMI was also positively correlated with parity and breast growth during pregnancy (Figure 6, Table A4 and Table A5).

Later onset of menarche was negatively correlated with breast growth during puberty and pregnancy. Breast growth during puberty was positively correlated with breast growth during pregnancy and negatively with parity. The duration of previous lactations was positively correlated with parity. Older mothers were less likely to use hormonal contraception, whilst mothers that had a vaginal birth were more likely to have a larger breast volume (Figure 6, Table A4 and Table A5).

## 4. Discussion

**Figure 7:**
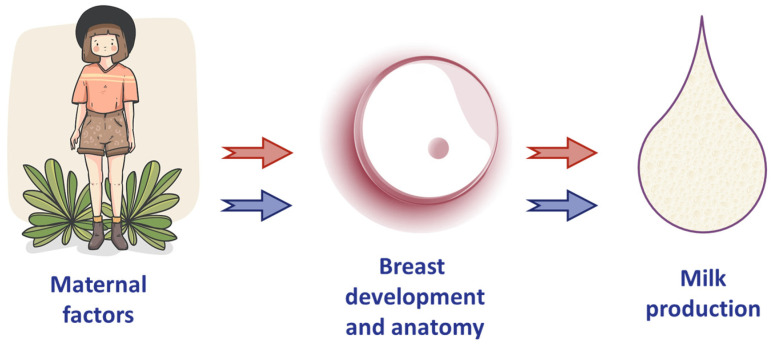
The findings of this study suggest that several modifiable and non-modifiable maternal factors may potentially lead to compromised breast development and impact milk production (red arrows). On the contrary, epigenetic changes from the first or previous lactations may positively impact the robustness of subsequent lactations (blue arrows).

This comprehensive pilot study shows that several modifiable and non-modifiable maternal factors including adiposity may potentially lead to compromised breast development and impact MP (Figure 7). Not surprisingly, the pathways culminating in either high or low MP start as early as puberty, a critical breast development window, with later menarche correlating with minimal pubertal and pregnancy breast growth. Diminished breast growth is further correlated with lower numbers and smaller diameters of the main milk ducts, which in turn correlated with a lower MP. Importantly, higher maternal adiposity also correlated with minimal breast growth during pregnancy and a lower 24 h MP.

### 4.1. Breast Growth During Puberty and Pregnancy

Whilst the human breast is preferably the sole source of infant nutrition during the first six months of life, studies of the lactating breast and its development are extremely rare [1]. The course of breast development consists of distinct phases starting at the fetal phase and advancing through the neonatal, prepubertal, and post-pubertal phases, after which further developmental stages are not achieved until pregnancy and lactation [50]. During pregnancy, breast secretory differentiation and proliferation occur under the influence of reproductive hormones, estrogen, progesterone, prolactin, and some metabolic hormones. After birth, triggered by the withdrawal of progesterone, secretory activation occurs to initiate copious milk secretion [51].

In our study, breast growth during puberty correlated with increased numbers and diameters of main milk ducts as well as with the mammary blood flow (internal mammary artery resistive index) during established lactation (Figure 3, Table A1 and Table A2). The breast growth during puberty is caused not only by the increased accretion of adipose tissue within the mammary gland but by a progressive elongation and branching of the milk ducts that creates a more extensive ductal network [52]. These changes coincide with the increase in plasma concentrations of estrogen, the luteinizing hormone, follicle stimulating hormone, growth hormone, and prolactin with each menstrual cycle [53,54]. Thus, it is not surprising that later menarche in our study was correlated with minimal breast growth not only during puberty but also in pregnancy, and that minimal growth during puberty was related to minimal breast growth during pregnancy. Furthermore, it may be possible to use an ultrasound to assess the composition of the breast, particularly in cases where no growth or minimal changes in density are experienced.

Indeed, during pregnancy, further extension and branching of the ductal system occurs, together with intensified lobular-alveolar growth (mammogenesis) influenced by multiple hormones, such as estrogen, progesterone, prolactin, growth hormone, insulin-like growth factor, epidermal growth factor, fibroblast growth factor, parathyroid hormone-related protein, and placental lactogen [55,56,57,58]. The proportion of growth (breast volume increase by bra cup size) during pregnancy varies widely between women, ranging from no increase to a considerable increase (192 ± 136 (−230–730) cm^3^) [26]. We have previously shown in a larger cohort (*n* = 609) that a larger pre-pregnancy breast volume and breast growth during pregnancy are associated with an increased MP [26]. Logically, in this study, breast growth during pregnancy was also positively correlated with the number of main milk ducts and their diameters as well as GTR (Figure 3, Table A1 and Table A2).

### 4.2. Breast Anatomy, Body Composition, and 24 h Milk Production

We did not observe any direct correlations of 24 h MP parameters with the onset of menarche, breast growth during puberty and pregnancy, nor with the number of main milk ducts or GTR. The latter two are in line with another smaller ultrasound study (*n* = 21) [28]. Likewise, the mean duct diameters did not correlate with the numbers of main milk ducts and were similar between the studies. However, in contrast, we observed multiple positive correlations of 24 h MP parameters, including breast storage capacity, with the mean diameters of the milk ducts (Figure 4, Table A3).

Sufficient volume of functional glandular tissue is a key requirement for successful lactation [27]. Whilst it is commonly believed that glandular tissue accounts for most of the breast volume during lactation, it has been shown that approximately one third of the breast tissue comprises adipose tissue, with great variability between lactating women [28]. Although no correlation of GTR with the 24 h MP was established in ours and the previous study [28], it is difficult to estimate the actual volume of glandular tissue in the breast due to its heterogenous nature. Whilst it is still unknown what is the healthy adiposity range for postpartum women, 39% of our participants were in the healthy range according to BMI, but only three participants (9%) were within a healthy adiposity range (<33%FM) when assessed by %FM [49]. Lower GTR was correlated with higher maternal body composition parameters, including FM and FFM and breast volume (Figure 3, Table A1). Further, increased breast volume was also related to higher maternal adiposity (Figure 5, Table A4) yet to minimal breast growth during pregnancy (Figure 3, Table A5), all of these highlighting the importance of healthy adiposity for lactation success.

The higher adipose tissue content in the breast with an increased BMI, which can be detected by ultrasound, may potentially impact several milk synthesis pathways. Leptin produced by the mammary gland adipocytes contributes to local proinflammatory mechanisms stimulating the production of inflammatory factors such as IL-1, IL-6, and TNF-α and enhances oxidative damage in the cells [59]. Additionally, a murine model study has shown that milk leptin adversely affects the MP in mammary epithelial cells, inhibiting casein production through the inactivation of STAT5, a transcriptional factor that induces MP [60]. Interestingly, women with higher adiposity have higher milk leptin concentrations [61], as their serum leptin concentrations are higher [62].

This evidence positions maternal adiposity as a central factor impacting MP on multiple levels. As such, we identified multiple negative correlations between maternal adiposity (FM, %FM, and FM/FFM) and 24 h MP parameters (Figure 4, Table A3), as well as breast growth during pregnancy (Figure 5, Table A5). To our knowledge, this is the first comprehensive study to link maternal body composition (adiposity) to MP in women. A recent systematic review noted a higher maternal BMI/adiposity was related to delayed secretory activation. However, it identified only six studies that analyzed BMI and MP, with half of the studies finding inverse associations between the two and the remaining failing to show any relationships; unfortunately, none of these studies measured maternal body composition [63]. Further, the analysis of 58 papers examining the relationship between maternal BMI (*n* = 28) and %FM (*n* = 30) and infant milk intake showed no associations. The review highlighted limitations such as methodological heterogeneity in acquiring the BMI/body composition data and the lack of BMI variability, specifically of the higher end of the range. In our study, despite the majority of participants having overweight or obesity, we also found no relationships between MP and BMI, although for BMI, there was a moderate negative correlation with GTR. To improve our understanding of the impact of maternal adiposity on MP, further studies should attempt to measure maternal body composition rather than BMI. Nevertheless, early interventions targeting healthy adiposity could improve breastfeeding outcomes, aligned with current public health recommendations [64].

Interestingly, milk duct characteristics, mammary blood flow, and milk removal parameters such as PAMR were not correlated with maternal body composition. This suggests the milk ejection reflex is intact, and that the anatomical composition of the breast does not impact the efficacy of milk removal (by the infant or the breast pump) in women with overweight/obesity or LMS. Thus, milk volume removed from the breast is likely limited by its synthetic and/or storage capacity rather than the mode of removal.

### 4.3. Mammary Blood Flow

Our study found that the internal mammary artery resistive index was positively correlated with both the breast growth during puberty and the return of the menstrual cycle but not 24 h MP (Figure 3, Table A1). Mammary blood flow doubles, on average, during pregnancy commensurate with an increasing mammary metabolism [29,65], yet it is rarely studied. Animal studies of mammary blood flow report either positive relationships [66,67] or no relationships with MP [68,69]. As LMS is one of the main reasons for the early cessation of breastfeeding [6,7], there is an urgent need for a more in-depth understanding of the factors that control milk synthesis in women. Given an adequate blood flow is required for the development of the mammary gland during pregnancy, it may be implicated in the suboptimal lactation outcomes observed more frequently in women with gestational hypertension and pre-eclampsia [70].

A single study of 55 women using different blood flow parameters also failed to find a relationship with the MP [30]. Despite the absence of a relationship, three women that had a low MP due to nipple piercing or hypoplasia had distinctly reduced mammary blood flow, indicating that milk synthesis might regulate mammary blood flow locally and that there may be a minimum threshold requirement of mammary blood flow for an adequate MP [30]. Furthermore, in our study, most of our participants had overweight or obesity, therefore, they were likely to have greater volumes of mammary adipose tissue. It is not possible to determine how much blood flow is required for the adipose tissue, which might potentially be masking the relationships with MP [30]. The Doppler ultrasound is therefore a promising diagnostic tool to investigate blood flow to the lactating breast.

### 4.4. Maternal Characteristics

We have also found several novel relationships between maternal characteristics, including reproductive and obstetric history, with breast anatomy and MP. Increased parity was related to higher numbers and diameters of the main milk ducts, as well as higher 24 h MP parameters including the maximum feed volume and breast storage capacity (Figure 4, Table A3). In contrast, lower parity as well as the reduced duration of previous lactations and lower maternal age were related to a greater GTR. Maternal age may impact the differentiation and proliferation of glandular tissue due to increased adiposity, decreased metabolism and increased insulin resistance [71], and the gradual degeneration of terminal duct lobular units [72]. Animal studies report greater milk yield with increasing parity [73,74]. Similarly, women report LMS more frequently in their first lactation [75], and a lower 24 h MP has been observed in primiparous compared to multiparous women [76,77], likely due to the earlier onset of secretory activation in multiparous mothers, as this difference was not detectable after the first week postpartum. Several other studies have shown no relationship between parity and infant milk intake [25,78,79,80]. Importantly, a recent longitudinal study showed no effect of consecutive lactations on the 24 h MP in fully breastfeeding women [13].

In this study, use of hormonal contraception during lactation was related to a greater GTR and 24 h MP (Figure 3 and Figure 4, Table A1 and Table A3). A recent scoping review [81] confirmed that progestin-only methods do not reduce MP but showed mixed evidence for estrogen-containing contraceptives, for which some studies show a decreased milk supply [9,81]. Whilst we do not have details on the type of hormonal contraception, it appears the MP was not impacted.

The return of menses was correlated with smaller milk duct diameters and a lower total and test breast 24 h MP (Figure 3 and Figure 4, Table A2 and Table A3). The menstrual cycle is known to affect the breast, with the highest mitotic activity during the luteal phase, resulting in the development of the lobules and alveoli, followed by the degeneration of this epithelial growth during the follicular phase [82,83]. Breastfeeding women have a longer postpartum period of amenorrhea and infertility than women who do not breastfeed [84]. However, it is not uncommon to experience cycle return whilst breastfeeding, with lower breastfeeding frequency relating to earlier postpartum cycle return [85]. Anecdotally, some women report a temporary or even a permanent drop in perceived milk supply in response to the re-commencement of menstruation. A small study has measured breast milk compositional changes related to increased epithelial cell permeability (increased sodium and chloride; decreased lactose and potassium) around the time of ovulation that could potentially be indicative of transient changes in MP [86]. Mechanistically reduced production at this time could be due to reduced osmotic pressure in response to lower lactose [87] and/or altered breastfeeding frequency in response to higher sodium/chloride concentrations in the milk.

The strengths of our study include the use of an ultrasound to assess breast anatomy and blood flow and the validated reference method to measure 24 h MP [88], in addition to milk sampling pre- and post-milk removal, allowing for the calculation of PAMR and breast storage capacity. Furthermore, we measured maternal body composition rather than relying on proxy measures of adiposity, such as BMI.

Our study does have several limitations. Whilst it is the largest study of this kind to date, it is a small pilot cross-sectional study of Australian women with mainly European ancestry and from a high socioeconomic setting; thus, the results cannot be generalized. We also collected self-reported bra size, as well as reproductive and obstetric history data, which may be affected by recall bias [89,90]; however, the self-reported age at menarche and prenatal and birth data are considered reliable [90,91,92]. Another limitation is the absence of comprehensive maternal metabolic health data covering the pre-pregnancy, pregnancy, and postnatal periods. We did not account for maternal stress, diet, or hormonal status; thus, our results may not be transferable to more diverse populations. The acute physical and mental stress can reduce the oxytocin release during a feed and impair the milk ejection reflex, potentially resulting in a reduced MP by preventing the emptying of the breast at each feed as seen in early postpartum [93,94] or by elevating levels of serum cortisol and decreasing insulin sensitivity, which are also associated with a lower MP [94]. However, we investigated the MP during established lactation, and the associations between maternal stress and breastfeeding outcomes in the later postpartum period are poorly understood, with studies failing to find relationships with anxiety or depressive symptoms [94].

Whilst maternal diet is known to affect human milk composition, particularly fatty acids [95,96] and amino acids [97], the MP volumes are only affected in cases of malnutrition or an unusually restrictive diet [98,99], and our sample was relatively homogenous and from a high socioeconomic setting where malnutrition is unlikely. The potential overnutrition in our participants has likely been addressed by the established relationships between maternal adiposity and MP. Further, the causes of suboptimal lactation outcomes in women with a high BMI/adiposity are likely complex, with higher rates of pregnancy, birth, and postpartum complications, maternal psychosocial factors, intended breastfeeding duration, weight bias from health care providers, and the physical challenges of breastfeeding, such as more difficulties with latch and positioning accompanying a larger body. Unfortunately, the longer the survey, the higher the risk of respondents’ fatigue, which may result in poor or inconsistent data; therefore, we were not able to address all aspects of maternal health and breastfeeding, such as stress and family breastfeeding history. Larger studies may address these factors.

## 5. Conclusions

Findings from this comprehensive study identify several modifiable and non-modifiable maternal factors, such as high adiposity and minimal breast growth during puberty and pregnancy that may potentially lead to compromised breast development and impact milk production. Ultrasound imaging represents a low-risk potential diagnostic tool to identify the low proportion of glandular tissue as a limiting factor for milk production. Further research may inform interventions aimed at maintaining healthy adiposity not only during pre-conception, pregnancy, and lactation but as early as childhood. Our results provide a rationale for the antenatal lactation assessment of women and timely interventions in those at an increased risk of low milk supply to ensure they reach their full lactation potential.

## Figures and Tables

**Table 1:** Maternal demographic and anthropometric characteristics.

Maternal Characteristics (*n* = 34)	Mean (SD) or *n* (%)
Age (years)	33.1 ± 4.6 ^1^
Highest education level	*n* = 31
High school	2 (6.5)
Certificate/apprenticeship	24 (77.4)
Tertiary degree	5 (16.1)
Race	*n* = 30
Australian	27 (90.0)
Asian	1 (3.3)
Other	2 (6.7)
Anhropometrics and BC with BIS	*n* = 33
BC measurement time (months postpartum)	4.0 ± 1.2
Weight (kg)	73.7 ± 12.7
Height (cm)	167.1 ± 6.7
Body mass index (kg/m^2^)	26.4 ± 4.6
Fat-free mass (kg)	45.4 ± 6.0
Fat-free mass index (kg/m^2^)	16.3 ± 2.1
Fat mass (kg)	28.3 ± 8.2
Fat mass index (kg/m^2^)	10.2 ± 3.0
Fat mass (%)	37.8 ± 5.4
Fat mass/fat-free mass ratio	0.62 ± 0.14
Breast volume (cm^3^)	791 ± 231

^1^ Data are mean ± standard deviation or *n* (%). BC, body composition; BIS, bioelectrical impedance spectroscopy.

**Table 2:** Participants’ reproductive and obstetric history.

Maternal and Infant Characteristics	Mean (SD) or *n* (%)
Maternal characteristics	*n* = 34
Parity (primiparous)	20 (58.8) ^1^
Previous lactation(s) duration (months) ^2^	24.7 ± 15.5
Age at menarche (years) ^3^	12.7 ± 1.7
Breast growth during puberty (yes) ^4^	16 (76.2)
Breast growth during pregnancy (yes)	31 (91.2)
Cycle commensed prior to study visit (yes)	6 (17.6)
Current use of hormonal contraception (yes)	7 (20.6)
Cycle commenced + hormonal contraception use (yes)	2 (5.9)
Birth details	*n* = 34
Birth gestation (weeks)	39.3 ± 1.0
Vaginal birth	19 (55.9)
Elective cesarean	10 (29.4)
Non-elective cesarean	5 (14.7)

^1^ Data are mean ± standard deviation or *n* (%). ^2^ *n* = 14; ^3^ *n* = 24; and ^4^ *n* = 21.

**Table 3:** Breast anatomy parameters.

Parameters (*n* = 34)	Mean (SD) or *n* (%)
Milk ducts’ measurements	*n* = 33
Total MD number	11.2 ± 3.0 ^1^
MD number in inferior half of the breast	7.8 ± 2.5
MD number in superior half of the breast	3.4 ± 1.8
Mean MD diameter ^2^	1.6 ± 0.5
Mean MD diameter in inferior half of the breast ^2^	1.6 ± 0.5
Mean MD diameter in superior half of the breast ^2^	1.9 ± 0.8
Sum of MD diameters	18.4 ± 7.0
Sum of MD diameters in inferior of the breast	12.4 ± 5.5
Sum of MD diameters in superior half of the breast	6.0 ± 3.4
Glandular tissue representation (GTR)	*n* = 34
Low (<30%)	5 (14.7)
Moderate (30–70%)	10 (29.4)
High (>70%)	19 (55.9)
Resistive index measurements	*n* = 19
IMA peak systolic velocity (cm/s)	39.3 ± 18.9
IMA end diastolic velocity (cm/s)	17.6 ± 9.3
IMA resistive index	1.3 ± 0.4

^1^ Data are mean ± standard deviation or *n* (%). MD, milk ducts; IMA, internal mammary artery. ^2^ *n* = 31.

**Table 4:** The 24 h milk production parameters.

Parametres (*n* = 33)	Mean (SD)
Total 24 h MP (g)	809 ± 235 ^1^
24 h MP, test breast ^2^ (g)	429 ± 144
Average feed, test breast (g)	76.0 ± 36.1
Maximum feed, test breast (g)	122.0 ± 54.3
Average PAMR, test breast (%)	62.8 ± 13.9
Breast storage capacity, test breast (g)	197.5 ± 78.7

^1^ Data are mean ± standard deviation. MP, milk production; PAMR, percentage of available milk removed. ^2^ Test breast was randomized at the start of the study visit.

## Data Availability

The data presented in this study are available on request from the corresponding author due to ethical reasons.

## Appendix A

**Table A1:** Correlations between maternal characteristics and breast anatomy (part 1).

Predictors	GTR		Internal Mammary Artery RI		Total MD Number		MD Number in Inferior HB		MD Number in Superior HB	
Parity	**−0.36 ^1^**	**0.039 ^2^**	−0.29	0.091	−0.07	0.71	−0.07	0.71	−0.02	0.92
Duration of previous lactations (months)	**−0.34**	**0.049**	−0.28	0.10	0.07	0.70	0.07	0.69	0.01	0.94
Time postpartum (months)	0.28	0.10	−0.11	0.54	−0.20	0.25	−0.22	0.21	−0.03	0.87
Maternal age (years)	**−0.35**	**0.040**	0.04	0.81	−0.11	0.54	−0.01	0.97	−0.17	0.33
Weight (kg)	**−0.65**	**<0.001**	−0.14	0.41	−0.02	0.90	0.12	0.50	−0.21	0.24
Body mass index (kg/m^2^)	**−0.63**	**<0.001**	0.02	0.92	0.07	0.68	0.24	0.18	−0.21	0.23
Fat-free mass (kg)	**−0.37**	**0.029**	−0.13	0.48	−0.09	0.59	0.10	0.56	−0.30	0.084
Fat-free mass index (kg/m^2^)	**−0.40**	**0.018**	0.07	0.69	0.02	0.89	0.26	0.14	−0.33	0.058
Fat mass (kg)	**−0.70**	**<0.001**	−0.15	0.39	0.03	0.85	0.11	0.54	−0.10	0.58
Fat mass index (kg/m^2^)	**−0.68**	**<0.001**	−0.04	0.81	0.10	0.58	0.18	0.30	−0.10	0.59
Fat mass (%)	**−0.74**	**<0.001**	−0.14	0.44	0.11	0.55	0.10	0.59	0.04	0.83
Fat mass to fat-free mass ratio	**−0.71**	**<0.001**	−0.14	0.42	0.11	0.54	0.09	0.60	0.05	0.79
Breast volume (cm^3^)	**−0.72**	**<0.001**	−0.07	0.70	−0.08	0.66	0.01	0.94	−0.15	0.39
Age at menarche (years)	−0.20	0.25	−0.27	0.12	−0.01	0.96	−0.04	0.80	0.04	0.82
Puberty breast growth (yes)	−0.04	0.82	**0.53**	**0.001**	**0.34**	**0.047**	**0.48**	**0.004**	−0.01	0.95
Pregnancy breast growth (yes)	**0.39**	**0.022**	0.19	0.28	**0.34**	**0.046**	0.04	0.81	**0.48**	**0.004**
Birth mode (vaginal)	−0.21	0.24	−0.25	0.15	0.17	0.34	−0.04	0.84	0.33	0.057
Cycle commenced (yes)	−0.09	0.63	**0.40**	**0.021**	−0.19	0.27	−0.31	0.079	0.11	0.53
Hormonal contraception use (yes)	**0.59**	**<0.001**	0.11	0.54	−0.17	0.34	−0.33	0.058	0.12	0.51

^1^ Data are correlation strength and ^2^ *p*-value. GTR, glandular tissue representation (low, medium, and high); HB, half of the breast (test breast randomized at the start of the study visit); MD, milk ducts; and RI, resistive index. Bold font indicates significant correlation.

**Table A2:** Correlations between maternal characteristics and breast anatomy (part 2).

Predictors	Mean MD Diameter		Mean MD Diameter in Inferior HB		Mean MD Diameter in Superior HB		Total Sum of MD Diameters		Sum of MD Diameters in Inferior HB		Sum of MD Diameters in Superior HB	
Parity	**0.57 ^1^**	**<0.001 ^2^**	**0.47**	**0.006**	**0.43**	**0.012**	**0.38**	**0.025**	0.28	0.11	**0.37**	**0.033**
Duration of previous lactations (months)	**0.53**	**0.001**	**0.39**	**0.022**	**0.49**	**0.004**	**0.44**	**0.009**	0.31	0.077	**0.41**	**0.016**
Time postpartum (months)	0.19	0.27	0.25	0.15	0.06	0.73	−0.03	0.85	−0.02	0.91	−0.04	0.84
Maternal age (years)	−0.01	0.98	−0.08	0.65	0.11	0.55	−0.08	0.66	−0.07	0.68	−0.04	0.80
Weight (kg)	0.01	0.94	−0.02	0.91	0.04	0.82	0.00	1.00	0.06	0.73	−0.10	0.58
Body mass index (kg/m^2^)	0.10	0.58	0.03	0.84	0.14	0.44	0.12	0.51	0.17	0.33	−0.05	0.80
Fat-free mass (kg)	0.14	0.43	0.12	0.50	0.15	0.39	0.06	0.75	0.14	0.42	−0.12	0.50
Fat-free mass index (kg/m^2^)	0.25	0.15	0.19	0.28	0.29	0.10	0.20	0.25	0.29	0.09	−0.07	0.70
Fat mass (kg)	−0.09	0.63	−0.12	0.50	−0.04	0.80	−0.04	0.82	−0.01	0.95	−0.06	0.72
Fat mass index (kg/m^2^)	−0.03	0.88	−0.08	0.65	0.02	0.93	0.04	0.83	0.06	0.72	−0.02	0.89
Fat mass (%)	−0.18	0.31	−0.20	0.25	−0.14	0.43	−0.07	0.69	−0.07	0.70	−0.03	0.86
Fat mass to fat-free mass ratio	−0.18	0.31	−0.21	0.22	−0.13	0.46	−0.07	0.71	−0.08	0.66	−0.01	0.96
Breast volume (cm^3^)	0.14	0.44	−0.05	0.77	0.29	0.092	0.01	0.94	−0.06	0.74	0.12	0.52
Age at menarche (years)	0.08	0.67	−0.01	0.96	0.11	0.54	0.13	0.46	0.02	0.93	0.15	0.38
Puberty breast growth (yes)	0.08	0.66	0.08	0.65	0.16	0.37	0.33	0.054	**0.49**	**0.003**	0.03	0.84
Pregnancy breast growth (yes)	0.07	0.70	0.07	0.71	0.03	0.86	**0.35**	**0.044**	0.17	0.35	**0.37**	**0.031**
Birth mode (vaginal)	0.10	0.58	−0.06	0.72	0.22	0.20	0.18	0.30	−0.07	0.71	**0.48**	**0.004**
Cycle commenced (yes)	**−0.41**	**0.016**	−0.30	0.085	**−0.70**	**<0.001**	−0.43	**0.010**	**−0.36**	**0.036**	−0.25	0.16
Hormonal contraception use (yes)	−0.09	0.63	−0.09	0.60	−0.05	0.80	−0.26	0.14	**−0.37**	**0.033**	0.02	0.90

^1^ Data are correlation strength and ^2^ *p*-value. HB, half of the test breast (test breast randomized at the start of the study visit); MD, milk ducts. Bold font indicates significant correlation.

**Table A3:** Correlations between maternal characteristics and breast anatomy and 24 h milk production parameters.

Predictors	Total 24 h MP		TB 24 h MP		TB Maximum Feed		TB Average Feed		TB PAMR		TB Storage Capacity	
Parity	**0.36 ^1^**	**0.039 ^2^**	0.31	0.070	**0.36**	**0.034**	0.32	0.068	0.02	0.89	0.39	**0.024**
Duration of previous lactations (months)	0.33	0.056	0.31	0.072	0.26	0.14	0.29	0.10	0.12	0.50	0.25	0.16
Time postpartum (months)	0.11	0.54	0.21	0.22	0.11	0.54	0.19	0.28	0.33	0.057	−0.03	0.88
Maternal age (years)	−0.10	0.58	−0.10	0.58	0.06	0.75	0.03	0.86	0.04	0.84	−0.03	0.87
Weight (kg)	−0.12	0.50	−0.14	0.43	−0.23	0.19	−0.19	0.27	−0.27	0.13	−0.07	0.71
Body mass index (kg/m^2^)	−0.15	0.39	−0.17	0.34	−0.20	0.27	−0.15	0.38	−0.18	0.31	−0.12	0.49
Fat-free mass (kg)	0.08	0.65	0.05	0.76	−0.01	0.95	0.08	0.63	−0.20	0.26	0.19	0.29
Fat-free mass index (kg/m^2^)	0.02	0.91	0.01	0.96	0.02	0.89	0.13	0.46	−0.10	0.59	0.11	0.52
Fat mass (kg)	−0.25	0.16	−0.26	0.14	**−0.36**	**0.039**	**−0.37**	**0.032**	−0.27	0.12	−0.24	0.17
Fat mass index (kg/m^2^)	−0.26	0.14	−0.27	0.12	−0.33	0.059	−0.34	0.050	−0.21	0.23	−0.27	0.12
Fat mass (%)	**−0.37**	**0.032**	**−0.34**	**0.049**	**−0.42**	**0.013**	**−0.50**	**0.003**	−0.23	0.19	**−0.40**	**0.021**
Fat mass to fat-free mass ratio	**−0.36**	**0.038**	**−0.36**	**0.037**	**−0.43**	**0.010**	**−0.50**	**0.002**	−0.23	0.20	**−0.41**	**0.015**
Breast volume (cm^3^)	−0.02	0.93	−0.14	0.44	−0.25	0.15	−0.20	0.26	−0.13	0.47	−0.02	0.92
Glandular tissue representation (low, medium, and high)	0.19	0.27	0.25	0.15	0.25	0.16	0.28	0.11	0.09	0.60	0.25	0.16
Total MD number	0.28	0.11	0.23	0.19	0.15	0.39	0.13	0.48	−0.07	0.71	0.18	0.30
MD number in inferior half	0.26	0.14	0.19	0.29	0.15	0.40	0.22	0.21	0.00	0.99	0.16	0.36
MD number in superior half	0.10	0.58	0.12	0.50	0.05	0.78	−0.10	0.58	−0.12	0.52	0.08	0.64
Total sum of MD diameters (mm)	**0.42**	**0.013**	**0.43**	**0.012**	**0.38**	**0.025**	**0.41**	**0.015**	0.17	0.35	0.33	0.056
Sum of MD diameters in inferior half (mm)	**0.35**	**0.046**	**0.41**	**0.017**	**0.39**	**0.022**	**0.47**	**0.005**	0.19	0.27	0.33	0.054
Sum of MD diameters in superior half (mm)	0.31	0.074	0.22	0.21	0.16	0.37	0.09	0.61	0.03	0.87	0.14	0.42
Mean MD diameter (mm)	**0.43**	**0.012**	**0.44**	**0.009**	**0.40**	**0.019**	**0.48**	**0.004**	0.33	0.053	0.30	0.087
Mean MD diameter in inferior half (mm)	**0.38**	**0.025**	**0.53**	**0.001**	**0.45**	**0.008**	**0.50**	**0.002**	0.30	0.084	**0.38**	**0.025**
Mean MD diameter in superior half (mm)	0.31	0.070	0.14	0.44	0.27	0.13	**0.39**	**0.021**	0.26	0.14	0.15	0.41
IMA resistive index	−0.02	0.91	−0.17	0.34	−0.07	0.70	0.02	0.93	0.30	0.081	−0.10	0.59
Age at menarche (years)	0.14	0.44	0.03	0.86	0.16	0.38	−0.05	0.79	0.13	0.46	0.02	0.92
Puberty breast growth (yes)	−0.02	0.93	−0.04	0.84	−0.29	0.093	−0.07	0.71	−0.05	0.79	−0.22	0.21
Pregnancy breast growth (yes)	0.12	0.51	−0.11	0.55	0.17	0.33	0.12	0.51	0.11	0.52	0.04	0.84
Birth mode (vaginal)	−0.06	0.73	0.00	1.00	−0.07	0.70	−0.02	0.90	−0.18	0.31	0.23	0.19
Cycle commenced (yes)	**−0.55**	**<0.001**	**−0.55**	**<0.001**	0.00	1.00	−0.11	0.53	0.04	0.84	0.06	0.75
Hormonal contraception use (yes)	**0.36**	**0.034**	0.28	0.11	−0.02	0.92	0.01	0.95	−0.16	0.38	0.29	0.10

^1^ Data are correlation strength and ^2^ *p*-value. MD, milk ducts; MP, milk production; PAMR, percentage of available milk removed; and TB, test breast, randomized at the start of the study visit. Bold font indicates significant correlation.

**Table A4:** Correlations between maternal characteristics and body composition parameters (part 1).

Predictors	Parity		Duration of Previous Lactations		Maternal Age		Breast Volume	
Parity	NA	NA	**1.00**	**<0.001**	0.30	0.089	0.26	0.14
Duration of previous lactations (months)	**1.00 ^1^**	**<0.001 ^2^**	NA	NA	0.33	0.053	0.26	0.14
Time postpartum (months)	−0.13	0.46	−0.17	0.34	−0.20	0.27	−0.14	0.43
Maternal age (years)	0.30	0.089	0.33	0.053	NA	NA	−0.04	0.82
Weight (kg)	0.25	0.15	0.15	0.39	−0.01	0.94	0.66	**<0.001**
Body mass index (kg/m^2^)	0.28	0.11	0.22	0.22	0.04	0.83	0.71	**<0.001**
Fat-free mass (kg)	0.30	0.084	0.13	0.45	−0.02	0.91	0.50	**0.003**
Fat-free mass index (kg/m^2^)	**0.35**	**0.042**	0.23	0.19	0.06	0.76	0.61	**<0.001**
Fat mass (kg)	0.18	0.31	0.14	0.44	−0.01	0.97	0.66	**<0.001**
Fat mass index (kg/m^2^)	0.20	0.27	0.18	0.32	0.02	0.91	0.68	**<0.001**
Fat mass (%)	0.09	0.63	0.12	0.51	0.04	0.81	0.51	**0.002**
Fat mass to fat-free mass ratio	0.08	0.65	0.11	0.52	0.02	0.89	0.53	**0.001**
Breast volume (cm^3^)	0.26	0.14	0.26	0.14	−0.04	0.82	NA	NA
Age at menarche (years)	0.08	0.63	0.00	0.98	0.02	0.90	0.24	0.17
Puberty breast growth (yes)	**−0.36**	**0.037**	−0.12	0.49	0.15	0.39	−0.03	0.85
Pregnancy breast growth (yes)	0.16	0.38	0.27	0.12	0.09	0.61	−0.55	**0.001**
Birth mode (vaginal)	0.19	0.28	0.19	0.29	−0.07	0.70	0.40	**0.018**
Cycle commenced (yes)	−0.03	0.89	−0.20	0.25	0.32	0.068	0.15	0.39
Hormonal contraception use (yes)	−0.28	0.11	−0.32	0.065	**−0.34**	**0.049**	−0.16	0.37

^1^ Data are correlation strength and ^2^ *p*-value. Bold font indicates significant correlation.

**Table A5:** Correlations between maternal characteristics and body composition parameters (part 2).

Predictors	Age at Menarche		Breast Growth During Puberty		Breast Growth During Pregnancy		Birth Mode (Vaginal)		Cycle Commenced		Hormonal Contraception Use	
Parity	0.08 ^1^	0.63 ^2^	**−0.36**	**0.037**	0.16	0.38	0.19	0.28	−0.03	0.89	−0.28	0.11
Duration of previous lactations (months)	0.00	0.98	−0.12	0.49	0.27	0.12	0.19	0.29	−0.20	0.25	−0.32	0.065
Time postpartum (months)	0.12	0.49	−0.25	0.15	−0.22	0.20	−0.30	0.081	−0.23	0.19	−0.17	0.34
Maternal age (years)	0.02	0.90	0.15	0.39	0.09	0.61	−0.07	0.70	0.32	0.068	**−0.34**	**0.049**
Weight (kg)	0.26	0.13	−0.12	0.49	**−0.66**	**<0.001**	0.03	0.86	−0.10	0.59	**−0.45**	**0.008**
Body mass index (kg/m^2^)	0.07	0.71	0.13	0.46	**−0.64**	**<0.001**	−0.03	0.88	0.03	0.85	**−0.57**	**<0.001**
Fat-free mass (kg)	0.16	0.37	0.04	0.84	**−0.60**	**<0.001**	0.03	0.85	−0.12	0.50	−0.25	0.15
Fat-free mass index (kg/m^2^)	−0.09	0.62	**0.39**	**0.022**	**−0.52**	**0.002**	−0.03	0.86	0.05	0.77	**−0.43**	**0.010**
Fat mass (kg)	0.31	0.079	−0.21	0.23	**−0.59**	**<0.001**	0.02	0.89	−0.06	0.75	**−0.56**	**0.001**
Fat mass index (kg/m^2^)	0.18	0.30	−0.07	0.71	**−0.60**	**<0.001**	−0.02	0.91	0.01	0.94	**−0.62**	**<0.001**
Fat mass (%)	0.24	0.16	−0.26	0.14	**−0.57**	**<0.001**	−0.06	0.73	0.05	0.76	**−0.51**	**0.002**
Fat mass to fat-free mass ratio	0.28	0.11	−0.27	0.12	**−0.52**	**0.001**	−0.03	0.86	0.02	0.91	**−0.56**	**0.001**
Breast volume (cm^3^)	0.24	0.17	−0.03	0.85	**−0.55**	**0.001**	**0.40**	**0.018**	0.15	0.39	−0.16	0.37
Age at menarche (years)	NA	NA	**−0.94**	**<0.001**	**−0.44**	**0.009**	0.32	0.065	−0.10	0.58	−0.14	0.42
Puberty breast growth (yes)	**−0.94**	**<0.001**	NA	NA	**0.40**	**0.020**	−0.29	0.095	0.09	0.61	0.19	0.29
Pregnancy breast growth (yes)	**−0.44**	**0.009**	**0.40**	**0.020**	NA	NA	−0.15	0.39	0.02	0.91	0.08	0.66
Birth mode (vaginal)	0.32	0.065	−0.29	0.095	−0.15	0.39	NA	NA	0.19	0.29	0.29	0.10
Cycle commenced (yes)	−0.10	0.58	0.09	0.61	0.02	0.91	0.19	0.29	NA	NA	0.27	0.12
Hormonal contraception use (yes)	−0.14	0.42	0.19	0.29	0.08	0.66	0.29	0.10	0.27	0.12	NA	NA

^1^ Data are correlation strength and ^2^ *p*-value. Bold font indicates significant correlation.

**Table A6:** Correlations between number of milk ducts, sum of duct diameters and mean duct diameters in the test breast.

Predictors	Total MD Number		MD Number in IH		MD Number in SH		Total Sum of MDD		Sum of MDD in IH		Sum of MDD in SH		Mean MDD		Mean MDD in IH		Mean MDD in SH	
Total MD number	NA	NA	**0.80** ^1^	**<0.001** ^2^	**0.54**	**0.001**	**0.66**	**<0.001**	**0.58**	**<0.001**	**0.41**	**0.016**	−0.03	0.88	0.05	0.77	−0.10	0.59
MD number in IH	**0.80**	**<0.001**	NA	NA	−0.07	0.69	**0.53**	**0.001**	**0.71**	**<0.001**	−0.06	0.73	−0.01	0.95	0.06	0.74	0.03	0.87
MD number in SH	**0.54**	**0.001**	−0.07	0.69	NA	NA	**0.35**	**0.041**	−0.03	0.87	**0.77**	**<0.001**	−0.03	0.87	0.00	0.98	−0.25	0.16
Total sum of MDD (mm)	**0.66**	**<0.001**	**0.53**	**0.001**	**0.35**	**0.041**	NA	NA	**0.88**	**<0.001**	**0.64**	**<0.001**	**0.71**	**<0.001**	**0.72**	**<0.001**	**0.48**	**0.004**
Sum of MDD in IH (mm)	**0.58**	**<0.001**	**0.71**	**<0.001**	−0.03	0.87	**0.88**	**<0.001**	NA	NA	0.19	0.27	**0.62**	**<0.001**	**0.72**	**<0.001**	**0.36**	**0.034**
Sum of MDD in SH (mm)	**0.41**	**0.016**	−0.06	0.73	**0.77**	**<0.001**	**0.64**	**<0.001**	0.19	0.27	NA	NA	**0.48**	**0.004**	0.33	0.059	**0.49**	**0.003**
Mean MDD (mm)	−0.03	0.88	−0.01	0.95	−0.03	0.87	**0.71**	**<0.001**	**0.62**	**<0.001**	**0.48**	**0.004**	NA	NA	**0.92**	**<0.001**	**0.76**	**<0.001**
Mean MDD in IH (mm)	0.05	0.77	0.06	0.74	0.00	0.98	**0.72**	**<0.001**	**0.72**	**<0.001**	0.33	0.059	**0.92**	**<0.001**	NA	NA	**0.47**	**0.005**
Mean MDD in SH (mm)	−0.10	0.59	0.03	0.87	−0.25	0.16	**0.48**	**0.004**	**0.36**	**0.034**	**0.49**	**0.003**	**0.76**	**<0.001**	**0.47**	**0.005**	NA	NA

^1^ Data are correlation strength and ^2^ *p*-value. IH, inferior half of the breast (test breast randomized at the start of the study visit); MD, milk ducts; MDD, milk duct diameter; NA, not applicable; and SH, superior half of the breast. Bold font indicates significant correlation.

**Table A7:** Correlations between 24 h milk production parameters.

Predictors	Total 24 h MP		24 h MP, TB		Maximum Feed, TB		Average Feed, TB		Average PAMR, TB		Breast Storage Capacity, TB	
Total 24 h MP (g)	NA	NA	**0.80** ^1^	**<0.001** ^2^	**0.54**	**<0.001**	**0.52**	**0.002**	0.23	0.19	**0.47**	**0.005**
24 h MP, test breast 2 (g)	**0.80**	**<0.001**	NA	NA	**0.58**	**<0.001**	**0.59**	**<0.001**	0.19	0.28	**0.54**	**<0.001**
Average feed, test breast (g)	**0.54**	**<0.001**	**0.58**	**<0.001**	NA	NA	**0.91**	**<0.001**	**0.39**	**0.024**	**0.69**	**<0.001**
Maximum feed, test breast (g)	**0.52**	**0.002**	**0.59**	**<0.001**	**0.91**	**<0.001**	NA	NA	**0.51**	**0.002**	**0.61**	**<0.001**
Average PAMR, test breast (%)	0.23	0.19	0.19	0.28	**0.39**	**0.024**	**0.51**	**0.002**	NA	NA	−0.23	0.19
Breast storage capacity, test breast (g)	**0.47**	**0.005**	**0.54**	**<0.001**	**0.69**	**<0.001**	**0.61**	**<0.001**	−0.23	0.19	NA	NA

^1^ Data are correlation strength and ^2^ *p*-value. MP, milk production; NA, not applicable; PAMR, percentage of available milk removed; and TB, test breast (randomized at the start of the study visit). Bold font indicates significant correlation.

**Table A8:** Correlations between maternal body composition parameters.

Predictors	Weight		BMI		FFM		FFMI		FM		FMI		%FM		FM/FFM		Breast Volume	
Weight (kg)	NA	NA	**0.80** ^1^	**<0.001** ^2^	**0.86**	**<0.001**	**0.76**	**<0.001**	**0.93**	**<0.001**	**0.85**	**<0.001**	**0.65**	**<0.001**	**0.68**	**<0.001**	**0.66**	**<0.001**
BMI (kg/m^2^)	**0.89**	**<0.001**	NA	NA	**0.68**	**<0.001**	**0.88**	**<0.001**	**0.88**	**<0.001**	**0.94**	**<0.001**	**0.71**	**<0.001**	**0.72**	**<0.001**	**0.71**	**<0.001**
FFM (kg)	**0.86**	**<0.001**	**0.68**	**<0.001**	NA	NA	**0.80**	**<0.001**	**0.60**	**<0.001**	**0.50**	**0.003**	0.18	0.31	0.21	0.24	**0.50**	**0.003**
FFMI (kg/m^2^)	**0.76**	**<0.001**	**0.88**	**<0.001**	**0.80**	**<0.001**	NA	NA	**0.59**	**<0.001**	**0.66**	**<0.001**	0.29	0.095	0.30	0.085	**0.61**	**<0.001**
FM (kg)	**0.93**	**<0.001**	**0.88**	**<0.001**	**0.60**	**<0.001**	**0.59**	**<0.001**	NA	NA	**0.96**	**<0.001**	**0.88**	**<0.001**	**0.90**	**<0.001**	**0.66**	**<0.001**
FMI (kg/m^2^)	**0.85**	**<0.001**	**0.94**	**<0.001**	**0.50**	**0.003**	**0.66**	**<0.001**	**0.96**	**<0.001**	NA	NA	**0.90**	**<0.001**	**0.91**	**<0.001**	**0.68**	**<0.001**
%FM (%)	**0.65**	**<0.001**	**0.71**	**<0.001**	0.18	0.31	0.29	0.095	**0.88**	**<0.001**	**0.90**	**<0.001**	NA	NA	**0.99**	**<0.001**	**0.51**	**0.002**
FM/FFM	**0.68**	**<0.001**	**0.72**	**<0.001**	0.21	0.24	0.30	0.085	**0.90**	**<0.001**	**0.91**	**<0.001**	**0.99**	**<0.001**	NA	NA	**0.53**	**0.001**
Breast volume (cm^3^)	**0.66**	**<0.001**	**0.71**	**<0.001**	**0.50**	**0.003**	**0.61**	**<0.001**	**0.66**	**<0.001**	**0.68**	**<0.001**	**0.51**	**0.002**	**0.53**	**0.001**	NA	NA

^1^ Data are correlation strength and ^2^ *p*-value. BMI, body mas index; FFM, fat-free mass; FFMI, fat-free mass index; FM, fat mass; FMI, fat mass index; FM/FFM, fat mass to fat-free mass ratio; NA, not applicable. Bold font indicates significant correlation.
